# Rating of personality disorder features in popular movie characters

**DOI:** 10.1186/1471-244X-5-45

**Published:** 2005-12-08

**Authors:** Morten Hesse, Sanna Schliewe, Rasmus R Thomsen

**Affiliations:** 1 Centre for Alcohol and Drug Research, Aarhus University, Købmagergade 26E, 1150 Copenhagen, Denmark; 2 Department of Psychology, University of Copenhagen, Øster Farimagsgade 5, 1353 København K

## Abstract

**Background:**

Tools for training professionals in rating personality disorders are few. We present one such tool: rating of fictional persons. However, before ratings of fictional persons can be useful, we need to know whether raters get the same results, when rating fictional characters.

**Method:**

Psychology students at the University of Copenhagen (N = 8) rated four different movie characters from four movies based on three systems: Global rating scales representing each of the 10 personality disorders in the DSM-IV, a criterion list of all criteria for all DSM-IV personality disorders in random order, and the Ten Item Personality Inventory for rating the five-factor model. Agreement was estimated based on intraclass-correlation.

**Results:**

Agreement for rating scales for personality disorders ranged from 0.04 to 0.54. For personality disorder features based on DSM-IV criteria, agreement ranged from 0.24 to 0.89, and agreement for the five-factor model ranged from 0.05 to 0.88. The largest multivariate effect was observed for criteria count followed by the TIPI, followed by rating scales. Raters experienced personality disorder criteria as the easiest, and global personality disorder scales as the most difficult, but with significant variation between movies.

**Conclusion:**

Psychology students with limited or no clinical experience can agree well on the personality traits of movie characters based on watching the movie. Rating movie characters may be a way to practice assessment of personality.

## Background

Personality disorders and personality traits represent a major challenge to many professionals dealing with psychiatric patients. Personality disorders impact treatment for substance use disorders [1,2], and mood disorders [3-6], bipolar disorder [7], and increase the risk of violence and other crime [8], as well as increase the risk of family conflict [9].

Precise and effective assessment of personality disorder is difficult, and the literature is fraught with research showing the difficulties in assessing personality disorders [10]. Most clinical psychologists make use of clinical observation and deduction based on behaviour in the clinic and clients narratives [11], and some researchers argue for the utility of clinical observations [12], and use clinical observations in studies of the psychometric properties of personality disorders [13,14]. Other researchers argue for the use of self-report instruments such as the Millon Clinical Multiaxial Inventory [15], or semi-structured interviews, such as the Structured Interview for the DSM-III-R Personality disorders [SIDP-R] [16], or the Structured Clinical Interview for the DSM-IV [SCID-II] [7]. Yet other researchers argue for the integration of information from a range of sources as the gold standard of personality disorder research [17,18].

Each of these approaches have their strengths and weaknesses, and may be appropriate in some situations, but not in others, depending on the purpose of assessment, client motivation, time and resources, or scientific questions in a research study [19]. However, in order for personality assessment to be clinically useful, mental health professionals working with patients must be able to understand and identify the various aspects of personality. Reading textbooks and hearing lectures on personality and personality disorder may provide basic knowledge, but future clinicians must also get a more specific idea of what personality assessment is. How do you observe the kinds of behaviour and patterns that are listed as criteria in the DSM-IV or the ICD-10? How do you evaluate such behaviours and integrate it into one or more diagnoses?

We suggest that one way to begin to learn personality observation may be through watching fictional movies and rate personality features based on observation of one or more main characters. This approach may be useful when practicing personality assessment in a situation where there are no real patients, such as when training medical students or psychology students. However, because there is no research to support that such observation is reliable, we present inter-rater agreement based on 4 movie characters.

## Method

### Procedure

Subjects were a convenience sample of psychology students (N = 8) at Copenhagen University who agreed to participate in a study of the inter-rater agreement of personality traits and personality disorders. Four were males, and four females. All had completed obligatory courses in personality psychology, psychiatry and clinical psychology.

Subjects came to the Centre for Alcohol and Drug Research on four nights one week apart. Two of the authors (S.S. and R.R.T.) led all participants into a common room, gave instructions, and started the movie. Subjects were instructed to rate the main character, and were informed about which character to focus on, before the movie started. At the end of the movie all participants went to separate rooms to complete the questionnaires about the movie on their own, and afterwards returned the questionnaires to the two authors.

After the end of the data collection procedure, the participants were invited to receive feedback about the results of the study.

### Instruments

Subjects rated the movie characters on three different personality measures: global rating scales for personality disorder representing each of the personality disorders listed in the DSM-IV [14,20], a list of the 79 criteria for the personality disorders listed in the DSM-IV in random order, and the Ten Item Personality Inventory [21].

#### Rating scales

The ten rating scales representing personality disorders have previously been used in two studies, one of inter-rater agreement of personality disorders [14], and one of convergent validity of personality disorders [20]. The rating scales range from 0 to 100, with scores from 0–29 representing the absence or very mild forms of the personality disorder, scores from 30–69 representing a moderate degree of the disorder, and scores of 70 or above represent marked presence. Each personality disorder is presented with three keywords as prompts.

The instruction was to circle the appropriate number to indicate the degree to which the character was similar to the personality disorder mentioned in each row.

#### The DSM-IV criteria

The 79 items of the DSM-IV were listed in random order to avoid halo bias, following the work of Blais and his colleagues [13]. Halo bias denotes the situation, where raters tend to be influenced by previous responses, so that for example if the rater has rated the first criterion for paranoid personality disorder as present, he or she will also be more likely to rate the presence of the second criterion. Each item was rated as (0) absent, [1] mildly or periodically present, or [2] present and causing significant distress. DSM-IV criteria count was calculated as the number of criteria rated as 2. The instructions indicated that subjects must read each sentence, and then circle a number from 0–2 to indicate the degree to which the criterion was true. Two criteria were changed: a brief description was given of the criterion of inadequate affect for schizotypal personality disorder, and a reference in the criterion of self-harm for personality disorder was changed, so that the exclusion of suicide attempts did not refer to another point in the list.

#### The Ten Item Personality Inventory [TIPI]

The TIPI is a very brief measure of the Big Five of personality: openness to experience, conscientiousness, extraversion, agreeableness, and neuroticism [21]. Each scale is measured by only to items rated on a 7 point Likert scale. Sample items are "1. Extraverted, enthusiastic. 2. Critical, quarrelsome." In the self-report version, the inventory instructs the respondent to "...write a number next to each statement to indicate the extent to which you agree or disagree with that statement. You should rate the extent to which the pair of traits applies to you, even if one characteristic applies more strongly than the other." This instruction was reworded to match the situation were a movie character was being rated.

The test-retest reliability and the convergent validity with other instruments of the TIPI has been reported to be good.

The Danish translation was made by two independent Danish translators, and retranslated into English by 2 different persons with English as their first language. Any observed differences were discussed and the final translation based on the feedback from the English-speaking translators.

#### Other information

Subjects also reported what courses they had finished in psychology of relevance (e.g., psychiatry, clinical psychology, personality psychology), and whether they had seen or heard about the film they were scheduled to see.

### Movies

Four movies were selected by the first author (M.H.). The movies were selected based on the following criteria:

• The personality and inner life of the main character must be important to the plot of the film.

• The main character should not undergo a complete transformation of personality (although some development in character was acceptable).

• The film should not directly be about psychiatric conditions or substance abuse, or represent a completely one-dimensional picture of a person (e.g., a comedy).

• The film should be in English, to allow for other researchers to attempt to replicate the findings.

The four movie characters that were selected were (in that order): Sarah Morton in Swimming Pool, directed by Francois Ozon, played by Charlotte Rampling; Aileen Wuornos in Monster, directed by Patty Jenkins, played by Charlize Theron; Suzanne Stone in To Die for, directed by Gus Van Sant, played by Nicole Kidman; and Coleman Silk in The Human Stain, directed by Robert Benton, played by Anthony Hopkins.

### Statistical analyses

Inter-rater agreement was calculated through random effects analysis of variance. Intraclass correlations were calculated as the proportion of variance unique to each movie character relative to the total variance in a given scale. This measure of agreement is equivalent to kappa in interpretation [22]. A limitation to the ICC is that it is highly affected by variance, because if the total variance is small, then the unique variance of each rated target must necessarily be even smaller. This is similar to the way that the kappa statistic is limited by low base-rates when calculating agreement.

Analysis of variance was used to assess the multivariate and univariate difference between characters, using the SPSS GLM multivariate ANOVA module. Both the movie character and the rater were entered as factors in the model, and the scales from each instrument were then entered as dependent variables in separate analysis. The interaction between the two was not entered (as that would have resulted in 32 cells with n = 1). Bonferroni adjustments were made for all p-values for ratings to adjust for family-wise type 1 error (with 28 tests of inter-rater reliability, 3 multivariate and 25 univariate, all p-values were multiplied by 28).

Differences in the experienced difficulty of rating the characters were also analyzed using analysis of variance. Contrast analysis was reported for linear trend, and Bonferroni post hoc comparisons of the difficulty of the movies.

Graphs and partial intraclass correlations were produced with STATISTICA for Windows, V. 6.0 [23], and ANOVA was calculated on SPSS for Windows v. 11.5 [24].

## Results

### Inter-rater agreement

The results of the analyses of inter-rater agreement are summarized in table 1, 2, 3.

**Table 1:** ANOVA tables and intra-class correlations for the TIPI

	**Degrees of Freedom**	**F-value**	**P-value**	**Eta**^**2**^	**Intraclass correlation**
**TIPI**					
Multivariate	15,47.33	12.92	0.000	0.777	-
Openness	3,21	1.54	0.999	0.181	0.05
Conscientiousness	3,21	22.04	0.000	0.759	0.68
Extraversion	3,21	65.49	0.000	0.903	0.88
Agreeableness	3,21	9.89	0.002	0.585	0.50
Neuroticism	3,21	6.83	0.240	0.494	0.37

**Notes: **F-values and p-values represent the effect of the character (i.e., the target being rated). TIPI: the Ten Item Personality Inventory [21]. All effects are controlled for between-rater variance. Partial intraclass correlation calculated based on intraclass correlations method 1 [22].

**Table 2:** ANOVA tables and intra-class correlations for the personality disorder rating scales

	**Degrees of Freedom**	**F-value**	**P-value**	**Eta**^**2**^	**Intraclass correlation**
Multivariate	30,35.89	4.17	0.001	0.770	-
Paranoid	3,28	3.30	0.999	0.306	0.20
Schizoid	3,28	5.73	0.151	0.446	0.36
Schizotypal	3,28	3.94	0.246	0.418	0.28
Antisocial	3,28	10.29	0.001	0.656	0.54
Borderline	3,28	7.55	0.016	0.557	0.46
Histrionic	3,28	10.56	0.006	0.600	0.54
Narcissistic	3,28	8.15	0.008	0.584	0.48
Avoidant	3,28	4.73	0.339	0.399	0.31
Dependent	3,28	1.15	0.999	0.167	0.04
Compulsive	3,21	8.08	0.015	0.561	0.47

**Notes: **F-values and p-values represent the effect of the character (i.e., the target being rated). Rating scales representing each personality disorder [14]. All effects are controlled for between-rater variance. Partial intraclass correlation calculated based on intraclass correlations method 1 [22].

**Table 3:** ANOVA tables and intra-class correlations for the personality disorder criteria counts

	**Degrees of Freedom**	**F-value**	**P-value**	**Eta**^**2**^	**Intraclass correlation**
Multivariate	30,35.90	14.62	0.000	0.920	-
Paranoid	3,21	4.08	0.999	0.368	0.24
Schizoid	3,21	6.19	0.999	0.469	0.56
Schizotypal	3,21	9.93	0.069	0.587	0.51
Antisocial	3,21	28.54	0.000	0.803	0.75
Borderline	3,21	19.04	0.006	0.731	0.65
Histrionic	3,21	24.73	0.001	0.779	0.72
Narcissistic	3,21	49.30	0.000	0.876	0.89
Avoidant	3,21	7.58	0.136	0.520	0.41
Dependent	3,21	14.11	0.025	0.668	0.62
Compulsive	3,21	15.11	0.016	0.683	0.63

**Notes: **F-values and p-values represent the effect of the character (i.e., the target being rated). Criteria rated in random order (after 13). All effects are controlled for between-rater variance. Partial intraclass correlation calculated based on intraclass correlations method 1 [22].

The rating scales for severity of personality disorders resulted in a significant overall model (F(30,35.89) = 4.17, p < 0.001, eta^2 ^= 0.770). The intraclass correlations ranked from poor agreement (all cluster A, and avoidant and dependent personality disorder, ICC ranging from 0.04 to 0.36) to moderate agreement (all cluster B and obsessive-compulsive personality disorder, ICC ranging from 0.46–0.54).

The five-factor model likewise resulted in a significant overall model (F(15,47.33) = 12.92, p < 0.001, eta^2 ^= 0.777). The intraclass correlations ranged from no agreement (openness to experience and neuroticism, ICC = 0.05 to 0.37) to excellent agreement (extraversion, ICC = 0.88).

Finally, when the DSM-IV criteria were used, the multivariate effect was 0.938 (F(30,32.96) = 17.83, p < 0.001). Criteria count for one personality disorder had poor agreement, paranoid personality disorder (ICC = 0.24). The remaining had either moderate agreement (schizoid, schizotypal, avoidant, dependent, obsessive-compulsive), or excellent agreement (antisocial, borderline, histrionic and narcissistic personality disorder criteria counts).

### The perceived difficulty of rating

We asked about the difficulty of rating each type of instrument for each film. Generally, we expected that the rating would become easier with each film. This was true of the perceived difficulty of the specific criteria for personality disorders, which declined significantly from film to film (estimate of linear trend = -0.53, 95% confidence interval [CI] = -0.95;-0.11). Sarah Morton in "Swimming Pool", the film seen first, was rated more difficult to rate on the criteria than those in the to last films (Suzanne Stone and Coleman Silk).

The TIPI, which was designed to be easy to understand and fill in, became more difficult to rate (estimate = 0.56, 95% CI = 0.14;0.91). The TIPI was perceived as easiest to rate for Sarah Morton from Swimming Pool, easier than Suzanne Stone or Aileen Wuornos (p < 0.05), and Suzanne Stone was rated more difficult than Coleman Silk (p < 0.05).

### Description of the movie characters

The descriptions of the movie characters in the following are based on the criteria count and the TIPI (see additional file 1 for descriptive statistics). Note that in terms of diagnoses, personality disorder diagnoses require satisfaction of half the criteria listed or more. The descriptions start with a very brief description of the characters as they are in the movies followed by description of the results of the ratings.

#### Sarah Morton [SM]

In the movie, "Swimming Pool", Sarah Morton is a writer of detective novels who is temporarily in a crisis. She agrees to borrow her publishers house in France for the summer to work there. While she lives there, his French-speaking daughter arrives and her promiscuous life-style both shocks and fascinates Sarah. Sarah does not appear to have many close relationships.

She was described in the ratings as the highest scorer on paranoid, schizoid, schizotypal, avoidant and obsessive-compulsive personality disorder of all the characters (see figure 2). She is not prototypical for any of these personality disorders, and only her score on schizoid would approach (but not reach) a diagnosis of personality disorder. In terms of the five-factor model, her profile is much more clear-cut: she is the most conscientious of all the characters, by far the least extroverted, quite disagreeable and a medium-high scorer on neuroticism.

Had this very introverted person been evaluated for personality disorder diagnosis and the shown symptom counts had been the result, she could either get a diagnosis of personality disorder not otherwise specified, or of not being personality disordered, depending on her clinical state at the time.

#### Aileen Wuornos [AW]

In the film "Monster", Aileen is a prostitute who falls in love with a young lesbian woman. Shortly after a man rapes her and tries to kill her, but she succeeds in killing him instead, and after that starts to kill men whom she contacts as a prostitute. Please note that the Aileen Wuornos described in this paragraph is the Aileen of the film as seen by the raters in this study – not the real character.

AW was perceived as a person with co-morbid borderline and antisocial personality disorder. Her scores on other personality disorders are well below diagnostic cut-offs. In terms of the five-factor model, she is less conscientious than the others, and a medium-high scorer on neuroticism. She would clearly be diagnosed with co-morbid borderline and antisocial personality disorder, had this been a diagnostic evaluation (indeed, the real Aileen Wuornos was diagnosed with borderline and antisocial personality disorder by clinicians who saw her) [25].

#### Suzanne Stone [SS]

Suzanne Stone in the film "To Die For" is a young woman who wants to be on television at any cost. She marries a young man, but soon begins to have affairs with TV producers to accomplish her main goal: to become a news-reporter at a major TV station. When her husband tries to persuade her to settle down and have children, she decides to have him killed instead, taking advantage of three troubled youths, whom she has met while trying to make a TV production.

SS was seen as a prototypical narcissistic person by the raters: on average, she satisfied 8 of 9 criteria for narcissistic personality disorder, some histrionic personality disorder criteria, and relatively few others.

In terms of the five-factor model, she is as open to experience as the others, as conscientious as the others (except for AW), as extraverted (except for SM), as disagreeable (except CS), and a low-scorer on neuroticism.

Had she been evaluated for personality disorders, she would receive a diagnosis of narcissistic personality disorder.

#### Coleman Silk [CS]

Coleman Silk in the movie "The Human Stain" is a middle-aged university professor who gets fired from his job under suspicion of racism against two African-American students, whom he has never met (and thus, he does not know the colour of their skin). The loss of his jobs leads to a chain of events that reveals much about his difficult life as well as lead him into new experiences. Among other things, it turns out that he has grown up in a family of African-Americans, and has kept this fact a secret for various reasons.

CS was rated as the least disordered of all the characters, scoring very low on all personality disorder scales. In terms of the five-factor model, he was seen as more agreeable than the others, and the lowest scorer on neuroticism.

Had he been evaluated for personality disorders, he would not be diagnosed with a personality disorder.

## Discussion

The findings overall indicate that various personality features of movie characters can be rated reliably by relatively untrained raters with only very basic knowledge about the nature of personality and personality disorders. It was found that the perceived difficulty of rating personality disorder criteria declined somewhat over the movies.

The use of specific criteria resulted in a better discrimination than the use of global rating scales, but even with global rating scales, agreement could be detected beyond chance, accounting for more the three-quarters of the observed variance.

In comparison with real-life patients, all raters were able to observe exactly the same behaviour for each target, yet the amount of situations and contexts that raters could observe were far more varied than would usually be available. In real-life settings, various clinicians will often have to observe patients within a single limited setting (e.g., counselling, therapy, group therapy, milieu therapy), and that setting may not even be the same for different clinicians (e.g., observing the patient on different days). However, rating movie characters also differ from another common situation, in which a co-interviewer rates criteria based on a semi-structured clinical interview. In that situation, inter-rater agreement is expectedly much higher than what was observed in this study. Thus, a strength of this study is to show that the inference of personality disorder traits can be done, even in the absence of clear questions about the specific criteria.

The rating of movie characters may serve as illustrations of many points in the assessment of personality disorders.

First of all, raters get the point that some people, whilst clearly disturbed and stressed as a result of life-long patterns of maladaptive behaviour, such as Aileen Wuornos and Sarah Morton, do not fit any single prototypical personality disorder profile [26-28].

Thirdly, raters get an impression of the relative precision (and lack thereof) of ratings of personality. Some students may perceive personality disorder diagnoses as nearly arbitrary or prejudice-driven labels, and the experience that agreement can be achieved may help them understand that there is something more than a label to personality disorders. Others with an unrealistic faith in the diagnostic system may experience that rating personality disorders is more difficult, and that although agreement is substantial, sometimes behaviours and reactions in the same person is experienced differently by different raters, even when using the same diagnostic system to rate the behaviours.

Several limitations to this study must be acknowledged. The four movies selected did not represent a wide range of different personality traits and personality disorders. For instance, the variance of the TIPI Openness to Experience scale is nearly nil, and there are no high scorers on avoidant or dependent personality disorder, either by the rating scales or by the criteria. Therefore, the level of agreement that could be reached for these traits and disorders is limited by the limited range they represent. Thus, although it is tempting to suggest that differences in agreement between features are due to difference between the degree to which behaviours are easily observable (e.g., conscientiousness, extraversion, Cluster B personality disorder feature), an equally justifiable interpretation is that the amount of variance for some personality traits (e.g., openness to experience, paranoid, schizoid, avoidant and dependent personality disorder) was simply too small to assure reasonable agreement.

Also, the difficulty in rating various areas must be interpreted with caution, given that all raters saw the films in the same order. Had the order been varied across raters, changes in perceived difficulty over time would have been much more reliable.

Thirdly, we do not know whether these raters were better or worse than the average psychology student at rating personality features. What we do know is that they did not consider themselves experts. The absence of a gold standard for the ratings makes it difficult to conclude anything about the movie characters, beyond simply stating that 8 independent raters reached similar results when rating these 4 movies.

Another limitation is the use of relatively untrained psychology students. With regard to agreement, however, it seems likely that experts could do little better in terms of cluster B personality disorder features, where it is unlikely that agreement can be better than ICC ranging from 0.46–0.54 for global rating scales, and ICC ranging from 0.75–0.89 for criteria counts.

A next step in evaluating the use of fiction as a tool for practicing assessment of personality disorder would be to conduct a study of the inter-rater agreement on real patients before and after practice with fiction, and identify factors in movies that foster the learning experience of rating personality features in movies. For instance, is it more helpful to assess ambiguous characters, easily rated characters, or a mixture of ambiguous or easily rated characters?

## Conclusion

Raters converge on their rating of personality traits in movie characters. Fiction movies may be useful for training observers in recognizing personality pathology and personality traits.

**Table 4:** Perceived difficulty by movie character

	**PD scales**		**PD criteria**		**TIPI**	
	**Mean**	**Standard deviation**	**Mean**	**Standard deviation**	**Mean**	**Standard deviation**

SM	3.10	0.74	3.00	0.67	1.50	1.08
AW	2.56	0.88	2.67	0.50	2.56	1.13
SS	2.80	0.92	2.00	0.82	2.80	0.79
CS	2.50	1.08	2.40	0.97	2.30	0.95
Total	2.74	0.91	2.51	0.82	2.28	1.07

Bonferroni Post hoc			SR>SS		SR<SS,AW	

**Notes: **PD: Personality disorder. Difficulty scores could ranged 0–4. SM: Sarah Morton. AW: Aileen Wuornos. SS: Suzanne Stone. CS: Coleman Silk

## Pre-publication history

The pre-publication history for this paper can be accessed here:



## Supplementary Material

Additional File 1 Descriptive statistics for the 4 characters. Click here for file
