# Supplementary material for: Rating of personality disorder features in popular movie characters
Source: BMC Psychiatry. 2005 Dec 8;5:45. doi: 10.1186/1471-244X-5-45 (PMC1325244; doi:10.1186/1471-244X-5-45)
Supplement: Additional File 1 — Descriptive statistics for the 4 characters. [file 1471-244X-5-45-S1.doc]

# Descriptive statistics for the 4 characters

|  | **Sarah Morton** | | **Suzanne Stone** | | **Aileen Wournos** | | **Coleman silk** | |
| --- | --- | --- | --- | --- | --- | --- | --- | --- |
|  | Mean | SD | Mean | SD | Mean | SD | Mean | SD |
| **Global rating scales** |  |  |  |  |  |  |  |  |
| Paranoid | 68.75 | 11.26 | 37.50 | 27.65 | 46.25 | 31.14 | 35.00 | 20.70 |
| Schizoid | 57.50 | 35.36 | 13.75 | 19.96 | 23.75 | 30.21 | 8.75 | 11.26 |
| Schizotypal | 67.50 | 31.05 | 27.50 | 31.05 | 47.50 | 38.08 | 18.75 | 21.67 |
| Antisocial | 30.00 | 33.81 | 78.75 | 23.57 | 73.75 | 29.25 | 16.25 | 22.00 |
| Borderline | 32.50 | 30.59 | 36.25 | 32.49 | 66.25 | 23.26 | 6.25 | 5.18 |
| Histrionic | 25.00 | 31.62 | 75.00 | 24.49 | 26.25 | 32.04 | 5.00 | 7.56 |
| Narcissistic | 42.50 | 30.59 | 86.88 | 21.87 | 38.75 | 40.51 | 17.50 | 16.69 |
| Avoidant | 51.25 | 35.63 | 2.50 | 4.63 | 23.75 | 30.68 | 21.25 | 22.32 |
| Dependent | 16.25 | 23.87 | 0.00 | 0.00 | 20.00 | 30.71 | 11.25 | 24.16 |
| Compulsive | 83.75 | 15.98 | 72.50 | 27.65 | 33.75 | 36.62 | 28.75 | 25.32 |
| **Criteria counts** |  |  |  |  |  |  |  |  |
| Paranoid | 2.63 | 1.60 | 0.75 | 1.16 | 2.00 | 1.69 | 0.75 | 1.16 |
| Schizoid | 3.38 | 1.60 | 2.25 | 1.04 | 1.88 | 1.89 | 0.25 | 0.71 |
| Schizotypal | 3.25 | 1.67 | 1.25 | 1.28 | 1.88 | 1.64 | 0.00 | 0.00 |
| Antisocial | 0.38 | 0.52 | 2.50 | 0.93 | 4.75 | 2.12 | 0.13 | 0.35 |
| Borderline | 1.75 | 1.04 | 0.63 | 0.74 | 5.13 | 2.30 | 0.38 | 0.74 |
| Histrionic | 0.88 | 0.83 | 3.88 | 1.13 | 1.75 | 1.04 | 0.00 | 0.00 |
| Narcissistic | 2.88 | 1.55 | 7.75 | 1.04 | 2.75 | 2.19 | 0.00 | 0.00 |
| Avoidant | 2.38 | 1.77 | 0.13 | 0.35 | 1.25 | 1.58 | 0.25 | 0.46 |
| Dependent | 0.00 | 0.00 | 0.25 | 0.71 | 1.75 | 0.89 | 0.25 | 0.46 |
| Compulsive | 3.13 | 1.81 | 1.88 | 0.64 | 0.38 | 0.52 | 0.25 | 0.46 |
| TIPI |  |  |  |  |  |  |  |  |
| Open. | 0.25 | 2.25 | 1.12 | 1.73 | 0.50 | 2.39 | 2.50 | 1.93 |
| Consc. | 5.25 | 1.39 | 2.63 | 2.50 | -2.57 | 4.12 | 4.75 | 0.89 |
| Extrav. | -5.13 | 0.64 | 4.00 | 1.20 | 1.50 | 1.85 | 2.88 | 1.96 |
| Agree. | -3.63 | 2.07 | -3.00 | 1.51 | -3.37 | 2.07 | 1.25 | 2.05 |
| Neurot. | 1.63 | 2.92 | -1.00 | 2.14 | 2.25 | 2.19 | -2.00 | 2.51 |
